# Microbe Profile: Salmonella Typhimurium: the master of the art of adaptation

**DOI:** 10.1099/mic.0.001521

**Published:** 2025-01-09

**Authors:** Blanca M. Perez-Sepulveda, Jay C. D. Hinton

**Affiliations:** 1 Clinical Infection, Microbiology & Immunology Department, Institute of Infection, Veterinary & Ecological Sciences, University of Liverpool, Liverpool, UK

**Keywords:** pathogen, *Salmonella*, genome, virulence

## Abstract

*Salmonella* Typhimurium is a major *Salmonella* serovar that is found globally. It is responsible for outbreaks of self-limiting gastroenteritis that are broadly linked to the industrialization of food production. *S*. Typhimurium is a pathogen with a broad host range and remarkable metabolic versatility. The ∼5 Mb genome includes the pSLT virulence plasmid and has a characteristic prophage repertoire. The major virulence determinants are encoded by a variety of pathogenicity islands. Emerging multidrug-resistant lineages of epidemics of *S*. Typhimurium are currently causing bloodstream infections in sub-Saharan Africa. The versatility and adaptability of *S*. Typhimurium pose an important public health challenge.

## Graphical abstract

Visual overview of the pathogenesis, virulence characteristics and taxonomic classification of *Salmonella* Typhimurium. Left panel: key characteristics of *Salmonella* Typhimurium. Right panel: taxonomy of *Salmonella enterica*, subspecies and serovars. iNTS: invasive non-typhoidal *Salmonella*; Mφ: macrophage; DC: dendritic cell; SPIs: *Salmonella* pathogenicity islands. Perez-Sepulveda & Hinton Microbe Profile: *Salmonella* Typhimurium.

## Taxonomy

*Salmonella enterica* subspecies *enterica* serovar Typhimurium (*S*. Typhimurium) was discovered by Theobald Smith in 1884 and named after his mentor Daniel Salmon. The genus *Salmonella* was officially adopted in 1934 and belongs to the family *Enterobacteriaceae*, order *Enterobacterales,* class *Gammaproteobacteria* and phylum *Pseudomonadota* (previously *Proteobacteria*). There are two species – *S. enterica* and *S. bongori*, of which the former is divided into six subspecies: subsp. I *enterica*, subsp. II *salamae,* subsp. IIIa *arizonae*, subsp. IIIb *diarizonae*, subsp. IV *houtenae* and subsp. VI *indica*. The serum agglutination serotyping scheme developed by Kauffmann and White has defined over 2600 *Salmonella* serovars. The serovar ‘Typhimurium’ reflects the original host animal (i.e. ‘*mouse* typhoid’ in Latin), described years before the Kauffmann–White scheme.

## Properties

*S*. Typhimurium is a rod-shaped, Gram-negative, facultative intracellular pathogen with a broad host range and an impressive metabolic versatility. The doubling time in a nutrient-rich medium at the optimal growth temperature of 37 °C is about 20 min. *S*. Typhimurium is unable to ferment lactose. The ability to utilize citrate as a sole carbon source is not shared by the related *S*. Typhi serovar. The metabolic adaptability of *S*. Typhimurium is exemplified by the ability of the strain LT2 to grow on at least 100 different compounds as sole carbon or nitrogen sources [1].

Protocols to enrich or select *S*. Typhimurium from a variety of sources, including faeces, are based on growth in the presence of agents such as selenite or tetrathionate which inhibit other bacterial species. Key biochemical properties include growth in selective media (brilliant green agar or xylose lysine deoxycholate) and fermentation of dextrose to produce H_2_S gas in the triple sugar iron test. The antigenic formula for *S*. Typhimurium is 1,4,[5],12:i:1,2, which specifies *S. enterica* subsp. I, possessing specific O antigens (4, 5 and 12) and phase 1 and 2 h (flagellar) antigens. Monophasic variants of *S*. Typhimurium lack the phase 2 h antigen (1,4,[5],12:i:-) and have distinct epidemiological characteristics. Conclusive identification of *S*. Typhimurium now involves molecular methods such as PCR or whole-genome sequencing.

## Genome

There are more genomes available for *Salmonella* than any other bacterial genus. Over 575 000 *Salmonella* genomes are available in EnteroBase (https://enterobase.warwick.ac.uk/species/index/senterica) of which approximately 70 000 were Typhimurium [accessed on 3 January 2025]. The genome of *S*. Typhimurium ranges from 4.5 to 5 Mb with a single circular chromosome and virulence plasmid, plus other plasmids. The first *S*. Typhimurium genome was sequenced in 2001 [2]. *S*. Typhimurium LT2 has a 4.9 Mb genome that encodes 4707 genes, including those on the 94 kb pSLT plasmid. About 70% of *S*. Typhimurium coding genes are shared with *E. coli* K12, which has aided the comprehensive annotation of other *Salmonella* genomes.

The accessory genome of *S*. Typhimurium is characterized by a distinct profile of plasmids, prophages and pathogenicity islands. The conjugative pSLT virulence plasmid belongs to the IncF incompatibility group and is carried by all *S*. Typhimurium isolates. This plasmid encodes the *spv* pathogenicity genes and shows lineage-based variations. *S*. Typhimurium has a diverse pool of prophages, with four functional prophages (Gifsy-1, Gifsy-2, Fels-1 and Fels-2) and four defective prophages (named Def1 to 4) identified in *S*. Typhimurium LT2. This prophage repertoire is commonly found in *S*. Typhimurium strains worldwide. Other *S*. Typhimurium prophages SopEφ, ST64B and P22 carry genes involved in virulence and other physiological processes, including *nanH, sodCI, sodCII, sopE, gogB* and *gtrABC. Salmonella* pathogenicity islands (SPIs) play a key role during mammalian infection, with SPI-1 to SPI-5 being required for *S*. Typhimurium virulence. Specifically, SPI-1 is required for invasion of epithelial cells and SPI-2 for intracellular replication in epithelial cells and macrophages.

## Phylogeny

Historically, subtyping of *Salmonella* was determined via susceptibility to a panel of phages (phage typing). However, genomics now offers the best tool for distinguishing pathovariants. Phage typing has been replaced by bioinformatic methods such as multilocus sequence typing (MLST) and single nucleotide variation analysis [3]. The *S*. Typhimurium phylogeny includes a range of pathovariants found in birds, animals, humans and food. Based on the core genome MLST, different lineages of *S*. Typhimurium have been identified that belong to particular sequence types (ST). *S*. Typhimurium ST19 has a broad host range and is associated with gastroenteritis in humans, whereas the host-restricted ST313 is linked to bloodstream infections in sub-Saharan Africa. Of all *S*. Typhimurium genomes available, 86% are ST19 and 7% are ST313 (EnteroBase). Epidemic strains of ST19 include several phage types, such as DT104 (primarily cattle), DT193/DT120 and U288 (primarily porcine), DT8 (ducks/geese) and DT160 (wild birds) [4]. Recently, another genome-based scheme has been developed for *Salmonella* using single nucleotide variations at different levels. The hierarchical clustering (HC) scheme that reflects serotype is at the level of 900 variations, with most *S*. Typhimurium isolates belonging to HC900_2 and a few strains designated as HC900_6511 and HC900_6910 [3].

Human bloodstream isolates of *S.* Typhimurium were found in Africa in the latter half of the 20th century, and belonged to a novel sequence type, ST313. The ST313 lineages L1, L2 and L3 differ by about 500 single nucleotide polymorphisms (SNPs) and can be distinguished from ST19 by 700 SNPs. ST313 isolates from the UK appear to represent an intermediate evolutionary step. Timed phylogenies have revealed the temporal accumulation of pseudogenes during the niche adaptation of *S*. Typhimurium. The dating of pseudogenes and prophage acquisition in *S*. Typhimurium ST313 shows that the most recent common ancestor (MRCA) of L1 is around 1794 (95% HPD AD 1738–1965), while the MRCA of L2 dates to about 1948 (95% HPD AD 1929–1959). The L3 lineage has the most recent MRCA, around 2007 (95% HPD AD 1998–2012) [5].

## Key features & discoveries

*S*. Typhimurium bacteria thrive in a wide variety of niches, including livestock, wild animals and the surface of plants. *S*. Typhimurium is mesophilic and can grow at a range of temperatures between 10 and 45 °C. The ability to survive and grow across a wide temperature range explains the persistence of the pathogen in remarkably diverse environments.

*Salmonella* is primarily transmitted to humans via the ingestion of contaminated food or through direct contact with infected individuals or animals. *S*. Typhimurium pathogenesis begins with adhesion to various surfaces on plants and in animals. The attachment of *S*. Typhimurium to mucosal surfaces in the mammalian intestine precedes invasion of epithelial cells and requires a range of adherent structures. In total, 12 adhesins have been discovered in *S*. Typhimurium, including chaperone-usher fimbriae, curli fimbriae, BapA and SiiE secreted by type 1 secretion systems, MisL, ShdA and SadA secreted by T5SS and potentially the PagN and Rck membrane proteins [6].

The interaction between *S*. Typhimurium and the mammalian host represents one of the most intensively studied infection models, involving both systemic and gut colonization of a wide range of animal species, including mice and birds [7]. We understand the molecular mechanisms underlying *Salmonella* invasion of cultured mammalian cells, including epithelial cells, dendritic cells and macrophages [7].

News reports often feature *S*. Typhimurium associated with foodborne human infections, emphasizing the importance of surveillance for public health and safety. Non-typhoidal *Salmonella* outbreaks are widely associated with gastroenteritis in humans, a zoonotic disease linked to the industrialization of food production, with *S*. Typhimurium being one of the main serovars isolated worldwide. *S*. Typhimurium has the ability to survive in conditions of low water activity, entering a dormant state that can allow the bacteria to survive for decades. Consequently, the pathogen has caused many foodborne outbreaks associated with low-moisture foods such as infant formula, chocolate and peanut butter, as well as in food processing environments.

Novel lineages of invasive non-typhoidal *Salmonella* (iNTS) pose a greater threat to human health with bloodstream infections causing about 77 000 deaths each year worldwide, mostly in sub-Saharan Africa. These *Salmonella* lineages are often multidrug resistant and target immunocompromised individuals, particularly children under 5 years of age. The S. Typhimurium ST313 lineages are responsible for a large proportion of human iNTS disease and began to cause high levels of bloodstream infections in Africa during the HIV epidemic of the 1980s [8].

The transcriptional landscape of *S*. Typhimurium has been studied intensively, identifying the precise regulation of genes involved in infection and the importance of post-transcriptional regulation mediated by small RNAs [9]. Key virulence determinants controlled at the transcriptional level include T3SSs, two-component regulatory systems, flagella and fimbriae. Expression of a wide variety of *S.* Typhimurium genes is modulated by the physico-chemical environment, including exposure of the bacteria to the acidity of the stomach or the anoxic gut milieu.

The complex regulatory mechanisms and impressive metabolic capacity of *S*. Typhimurium have profound implications for human health and food safety. *S*. Typhimurium has adapted to utilize a plethora of carbon sources and other nutrients [10] and can exploit metabolites produced by the gut microbiota to outcompete other pathogens during gastrointestinal colonization. The adaptability and versatility of *S*. Typhimurium are highlighted by various survival mechanisms, including the formation of persister cells that tolerate antimicrobial treatment and act as a reservoir of plasmid-encoded antimicrobial resistance genes. The urgent need to control the threat of S. Typhimurium requires focused efforts by microbiological researchers and infection biologists. It is hoped that an improved understanding of the intricate interplay of *Salmonella* gene function that leads to human disease will lead to innovative therapeutic interventions.

## Open questions

- How do alterations in microbiome composition affect *Salmonella* Typhimurium colonization and pathogenesis in humans?
- What is the relevance of single bacterial cell variation for *S*. Typhimurium virulence?
- How do host environmental conditions drive bacterial heterogeneity at the phenotypic level during infection?
- Is it possible to empirically demonstrate that *S.* Typhimurium ST313 has undergone adaptation to the human host?
- How are novel *S*. Typhimurium lineages transmitted in Africa?
- How will climate change impact the epidemiology of *S*. Typhimurium infections in humans?

## Abbreviations

HC: hierarchical clustering
HDP: highest posterior density
iNTS: invasive non-typhoidal *Salmonella*
MLST: multilocus sequence typing
MRCA: most recent common ancestor
NTS: non-typhoidal *Salmonella*
SNPs: single nucleotide polymorphisms
SPIs: *Salmonella* pathogenicity islands
sRNAs: small RNAs
ST: sequence types
TSI: triple sugar iron
T1SS: type 1 secretion systems
T3SS: type 3 secretion system
XLD: xylose lysine deoxycholate
